# Extracellular Vesicles in Microbes, Pathogens, and Infectious Diseases

**DOI:** 10.3390/ijms241310686

**Published:** 2023-06-26

**Authors:** Franklin Wang-Ngai Chow, Russell M. Morphew

**Affiliations:** 1 Department of Health Technology and Informatics, The Hong Kong Polytechnic University, Hung Hom, Hong Kong SAR, China; 2 Department of Life Sciences, Aberystwyth University, Aberystwyth SY23 3DS, UK

Extracellular vesicles (EVs) are nanosized lipid bilayer particles that are produced by all kinds of organisms, including both pathogenic and non-pathogenic archaea, bacteria, fungi, and parasites. These vesicles serve as a means of cell-free intercellular communication, playing a variety of roles including in quorum sensing or tuning microenvironments to benefit the survival of microbes. Importantly in pathogens, EVs can modulate host immune responses to evade elimination from the host. Moreover, besides facilitating the survival of pathogens and acting as a decoy to chemotherapeutics as a drug resistance mechanism, microbial EVs have potential applications in the development of therapeutics and diagnostics for infectious diseases. Furthermore, EVs can be utilised as vaccine candidates, for drug targeting, and for RNAi communication vehicles, aside from their potential as biomarkers for disease diagnostics.

The aim of this Special Issue is to feature the diverse roles and mechanisms of microbial EVs in relation to virulence, antibiotic resistance, and infectious diseases. Through the exploration of these topics, this issue aims to advance our understanding of the complex interplay between microbes and their hosts.

The quality control (QC) of EVs is highly topical and essential in research to ensure the accuracy and reliability of data, as well as to prevent bias and the misinterpretation of results. Steć et al., highlighted the importance of QC for Pectobacterium zantedeschiae EVs. They compared different purification and characterization methods identifying a good correlation between quantitative results incorporating an iodixanol density gradient ultracentrifugation purification method. Capillary electrophoresis was more selective and able to differentiate EV subpopulations, whilst nanoparticle tracking analysis (NTA) was unreliable due to impurities [1]. In addition, membrane vesicles of Pectobacterium have been found to play an important role in host–pathogen interactions and niche competition with other bacteria. Importantly, the mechanism of membrane vesicle production was also investigated, revealing the potential of membrane vesicles to act as a cargo delivery and secretion system [2].

In parasites, a laser microdissection analysis of the gastrodermal cells from the liver fluke, Fasciola hepatica, demonstrated that these cells are the primary source of secreted proteins and gut-derived EVs, which are released through a novel atypical secretory route. The authors suggest that gastrodermal cells play a crucial role in nutrient acquisition and potentially even immunomodulation by the parasite [3]. Additional parasitology-based EV research has focused on EVs derived from Plasmodium falciparum-infected red blood cells (RBCs) which were found to induce CD14+ CD16+ M2-like monocyte polarization, especially for those EVs produced from a severe malaria strain [4]. On the other hand, in viral infections, EVs from Chikungunya virus (CHIKV)-infected epithelial cells were found to encapsulate viral RNA and proteins and play a role in CHIKV transmission [5].

Antibiotic resistance is a severe threat to one’s health, and bacterial membrane vesicles (BMVs) have been identified as a potential target for combating antibiotic resistance. The multiple mechanisms that BMVs use to promote and mediate antibiotic resistance were reviewed by Liu X. et al. [6]. Studies have effectively shown that BMVs can transfer antibiotic resistance genes, sequester antibiotics, and facilitate biofilm formation. Consequently, BMVs could be investigated as conceptually new antibiotics and drug-delivery vehicles.

In human diseases, Rodriguez-Diaz and colleagues employed a next-generation sequencing (NGS) metagenomic approach to analyse the composition of faecal-microbe-derived EVs and their effect on the cellular permeability of intestinal cells in multiple diseases, including diarrhoea, morbid obesity, and Crohn’s disease. Their findings demonstrated that EVs derived from Bacteroidales and Pseudomonas were increased in the disease group compared to healthy controls. Moreover, EVs from these diseases were found to increase the permeability of CaCo-2 cells. These results provide insights into the potential role of EVs in the microbiome of these diseases and may have future therapeutic implications [7].

In summary, the studies in this Special Issue highlight the diverse roles and mechanisms of extracellular and membrane vesicles in disease pathology, virulence, and antibiotic resistance [1,2,3,4,5,6,7]. Understanding the composition, function, and production of these vesicles holds great potential for developing effective therapies and strategies for disease prevention and treatment.
